# Common origin of plasmid encoded alpha-hemolysin genes in *Escherichia coli*

**DOI:** 10.1186/1471-2180-10-193

**Published:** 2010-07-19

**Authors:** Ylanna Burgos, Lothar Beutin

**Affiliations:** 1 National Reference Laboratory for E. coli, Federal Institute for Risk Assessment (BfR), D12277 Berlin, Germany

## Abstract

**Background:**

Alpha (α)-hemolysin is a pore forming cytolysin and serves as a virulence factor in intestinal and extraintestinal pathogenic strains of *E. coli*. It was suggested that the genes encoding α-hemolysin (*hlyCABD*) which can be found on the chromosome and plasmid, were acquired through horizontal gene transfer. Plasmid-encoded α-*hly *is associated with certain enterotoxigenic (ETEC), shigatoxigenic (STEC) and enteropathogenic *E. coli *(EPEC) strains. In uropathogenic *E. coli *(UPEC), the α-*hly *genes are located on chromosomal pathogenicity islands. Previous work suggested that plasmid and chromosomally encoded α-*hly *may have evolved independently. This was explored in our study.

**Results:**

We have investigated 11 α-*hly *plasmids from animal and human ETEC, STEC and EPEC strains. The size of α-*hly *plasmids ranges from 48-157 kb and eight plasmids are conjugative. The regulatory gene (*hlyR*) located upstream of the *hlyCABD *gene operon and an IS*911 *element located downstream of *hlyD *are conserved. Chromosomally-encoded α-*hly *operons lack the *hlyR *and IS*911 *elements. The DNA sequence of *hlyC *and *hlyA *divided the plasmid- and chromosomally-encoded α-hemolysins into two clusters. The plasmid-encoded α-*hly *genes could be further divided into three groups based on the insertion of IS*1 *and IS*2 *in the regulatory region upstream of the α-*hly *operon. Transcription of the *hlyA *gene was higher than the housekeeping *icdA *gene in all strains (rq 4.8 to 143.2). Nucleotide sequence analysis of a chromosomally located α-*hly *determinant in *Enterobacter cloacae *strain indicates that it originates from an *E. coli *α-*hly *plasmid.

**Conclusion:**

Our data indicate that plasmids encoding α-*hly *in *E. coli *descended from a common ancestor independent of the plasmid size and the origin of the strains. Conjugative plasmids could contribute to the spread of the α-*hly *determinant to *Enterobacter cloacae*. The presence of IS-elements flanking the plasmid-encoded α-*hly *indicate that they might be mobile genetic elements.

## Background

Two major types of calcium dependent, pore forming cytolysins of the repeats in toxin (RTX)-family, called alpha-(α) and EHEC-hemolysin (enterohemolysin) were described in strains of *Escherichia coli *[1,2]. Both types of hemolysins are encoded by polycistronic operons consisting of four genes arranged in the order of *hlyCABD *[3,4]. The product of the *hlyC *gene is involved in activation of the hemolytic toxin the product of the *hlyA *gene. The gene products of *hlyB *and *hlyD *together with TolC are involved in secretion of the hemolysin through the bacterial cell wall [5].

EHEC-hemolysin is encoded on non-conjugative plasmids in strains of enterohemorrhagic *E. coli *(EHEC) that cause hemorrhagic diseases in humans [6,7]. In contrast, α-hemolysin is frequently associated with human uropathogenic *E. coli *(UPEC) strains [8] and with enterotoxigenic (ETEC), shigatoxigenic (STEC) and enteropathogenic *E. coli *(EPEC) strains that cause diarrhea and edema disease in animals [9-12].

In UPEC the α-*hly *genes are found on large chromosomal pathogenicity islands (PAI) [13,14]. The UPEC O4 (J96) and O6 (536) strains carry each two α-*hly *operons located on different PAIs [15,16], which contain divers junctions and adjacent sequences. This suggests that these loci have evolved independently [16,17]. Genetic analysis of chromosomal α-*hly *operons revealed differences in 5' flanking sequences and toxin expression [18-20].

Plasmid-encoded α-*hly *genes were found associated with EPEC O26 strains [21], as well as with ETEC and Shiga toxin 2e (Stx2e) producing STEC strains [9,10,22]. α-*hly *plasmids of *E. coli *were found to differ widely in size, incompatibility groups and conjugational transfer ability [10,20,21,23]. So far, only two plasmid α-*hly *operons were completely sequenced. The first is located on the 48 kb non-conjugative plasmid pHly152 from a murine *E. coli *strain [24]. The other is located on the 157 kb conjugative plasmid pEO5 of a human EPEC O26 strain [21]. Interestingly, despite the differences between pHly152 and pEO5, the DNA sequence of their α-*hly *operons are 99.2% similar while the sequence of the upstream regulatory *hlyR *region is 98.8% similar [21]. Importantly, the plasmid-inherited α-*hly *are less similar (96.0-96.4%) to the chromosomally inherited α-*hlyCABD *located on PAI I [GenBank AJ488511] and PAI II [GenBank AJ494981] of the *E. coli *strain 536 [18,21]. Moreover, chromosomally and plasmid-inherited α-*hly *operons also differ also for their 5' regulatory *hlyR *region. These findings suggest that the plasmid and chromosomal α-*hly *operons have evolved in parallel.

Studies on hemolysins of other bacterial species revealed similarities between the *E. coli *α-hemolysin genes and the *Enterobacter*, *Proteus, Morganella *and *Mannheimia *operons [25,26]. Codon usages base composition studies suggested that the α-*hlyCABD *genes of *E. coli *were originated from *Proteus, Morganella *or *Mannheimia *species [25,27]. Transposon-like structures found in the neighborhood of plasmid pHly152 and pEO5 encoded α-*hly *operons suggest that these were acquired by horizontal gene transfer [20,21].

The fact that the α-*hlyCABD *genes and their adjacent regions on pHly152 and pEO5 were highly similar to each other prompted us to investigate the genetic relationship between plasmid and chromosomal inherited α-*hly *operons in more strains of *E. coli *and in *Enterobacter cloacae*. Our results indicate that plasmid α-*hly *operons are highly similar regardless of differences in the plasmid backbone sequences, bacterial host and their source, suggesting that they have evolved from a common origin.

## Results

### Characterization of α-*hly *plasmids in *E. coli *strains

Nucleotide sequence analysis of the pEO5 α-*hlyCABD *operon and its 5' region revealed high similarity to the corresponding DNA segment of the α-*hly *plasmid pHly152 [21]. Since pEO5 and pHly152 differ in their origin, size and conjugative transfer, we investigated if plasmid α-*hly *operons have a common origin and evolved independently of chromosomal α-*hlyCABD *genes in *E. coli*.

**Figure 1:**
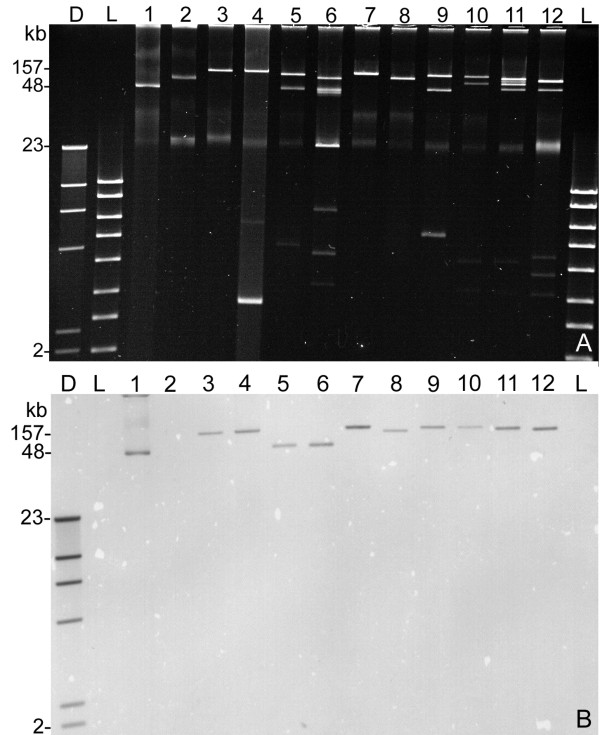
**Detection of plasmid encoded α-*hly *genes in *E. coli *strains**. A) Agarose gel (0.7%) with plasmid preparations obtained from *E. coli *strains. Lanes: D = digoxigenin-labelled molecular weight standard II (Roche); L = molecular weight standard hyperladder I (Bioline); 1 = 374 (phly152); 2 = TPE1313 (pO157); 3 = TPE422 (pEO5); 4 = TPE1030 (pEO5); 5 = 84-3208 (pEO11); 6 = 84-2573 (pEO12); 7 = 84-2195 (pEO9); 8 = 84-2 S (pEO14); 9 = 84-R (pEO13); 10 = CB853 (pEO853); 11 = CB855 (pEO855); 12 = CB857 (pEO857). B) Southern hybridization patterns of plasmid DNA from lanes 1-12 with the α-*hlyA *specific digoxigenin labelled gene probe generated with primers 10f/r from plasmid pEO5 DNA. The size of hybridizing α-*hly *plasmids varies from 48 (lane 1) to 157 kb (lane 3).

In order to explore the genetic relationship between plasmid α-*hly *genes we investigated five α-*hly *plasmids originating from canine ETEC strains and four plasmids of porcine ETEC and STEC strains (Table 1). α-hemolysin plasmids were detected by DNA-hybridization of Southern blotted plasmid DNA as described in Material and Methods (Fig. 1). The size of α-*hly *plasmids from dogs, pigs, mouse, cattle and human origin varied between 48 kb to 157 kb and other than pEO13, pEO14 and pEO860 all other plasmids were found transferable by conjugation (Table 1). Plasmid profile analysis has shown that the α-*hly*-plasmids are frequently found together with other large plasmids (Fig. 1).

**Table 1:** Relevant properties of strains carrying plasmid and chromosomally encoded α-*hly *determinants

					PCR products with primers pairs^a^					
**strain**	**Serotype^b^**	**Origin, reference^d^**	***hly*-plasmid (kb)**	**Plasmid group**	**1f/r** **(678 bp)**	**32f/r** **(671 bp)**	**44f/r** **(685 bp)**	**99f/r** **(650 bp)**	**72f/r** **(695 bp)**	**81f/r** **(773 bp)**

C4115	O26:[H11]	human, EPEC [21]	pEO5 (157)	1	+	+	+	+	-	-
TPE422	Or:H48	*E*. coli K12 (pEO5) [21]	pEO5 (157)	1	+	+	+	+	-	-
CB9866	O26:[H11]	cattle, EPEC [21]	pEO5 (157)	1	+	+	+	+	-	-
CB1027	O26:[H11]	human, EPEC [21]	pEO5 (157)	1	+	+	+	+	-	-
CB1030	O26:[H11]	human, EPEC [21]	pEO5 (157)	1	+	+	+	+	-	-
IP187	O26:[H11]	human, EPEC [21]	pEO5 (157)	1	+	+	+	+	-	-
84/2195	Ont:H10	dog [10]	pEO9 (146)	1	+	+	+	+	-	-
84-R	O121:H46	dog [10]	pEO13 (97)	1	+	+	+	+	-	-
374	Or:H48	mouse [24]	pHly152 (48)	2	+	e)	+	+	-	-
84-3208	O42:H37	dog, ETEC[10]	pEO11 (48)	2	+	e)	+	+	-	-
84-2573	O70:NM	dog, ETEC [10]	pEO12 (48)	2	+	e)	+	+	-	-
CB853	O138:H14	pig, STEC [29]	pEO853 (145)	3	+	f)	g)	+	-	-
CB855	O138:NM	pig, STEC [29]	pEO855 (140)	3	+	f)	g)	+	-	-
CB857	O157:NM	pig, ETEC [42]	pEO857 (97)	3	+	f)	g)	+	-	-
CB860	O149:H10	pig, ETEC [42]	pEO860 (48)	single	+	+	g)	+	-	-
84-2S	O75:H2	dog [10]	pEO14 (97)	single	-	-	-	-	-	-
536^h^	O6:K15:H31	human UPEC [20]	-	n.a	-	-	-	-	+	+
536-14	O6:K15:H31	PAI I deletion mutant of 536 [20]	-	n.a	-	-	-	-	+	-
695/83	O126:H27	human [19]	-	n.a	-	-	-	-	-	i)
J96^h^	O4:K6	human UPEC [46]	-	n.a	-	-	-	-	+	j)
KK6-16	*E. cloacae*	human [26]	-	n.a	k)	-	-	-	-	-

a) primer pairs and size of the PCR products obtained with strains TPE422 (pEO5) (primers 1f/r, 32f/r and 44f/r) and 536 (primers 81f/r and 72f/r) (see Table 2).

+ = a PCR product of the same size as obtained with strains TPE422 (pEO5) or 536, respectively.

- = no PCR product obtained

PCR products with other sizes than obtained with the reference strains are indicated for their length in bp.

b) O:H serotype, Or = rough LPS, Ont = O-antigen not typable, NM = non motile

c) α = alpha-hemolytic (α-*hly *genes), e-hly = enterohemolytic (EHEC-*hly *genes)

d) All strains were from feces if not otherwise indicated, ETEC = enterotoxigenic *E. coli*, STEC = shigatoxigenic *E. coli*, UPEC = uropathogenic *E. Coli*

e) 2000 bp PCR product (2007 bp as calculated from the nucleotide sequence of pEO11 [GenBank FM210249]

f) 2900 bp PCR product (2868 bp as calculated from the nucleotide sequence of pEO860) [GenBank FM210351]

g) 1500 bp PCR products

h) strain carries two α-*hly *determinants in the chromosome

i) 950 bp PCR product

j) 860 bp PCR product

k) 778 bp PCR product (calculated from nucleotide sequencing of KK6-16 [GenBank FM210352])

In addition, we investigated four *E. coli *and an *E. cloacae *strain with chromosomal α-*hly *operons (Table 1). A BLAST search using pEO5 [GeneBank FM180012] and phly152 [GeneBank M14107] sequences between *hlyR *and *hlyC *and downstream of *hlyD *revealed no similarity with sequences of chromosomal α-*hly *genes in strains CFT073 [GeneBank AE014075], UTI89 [CP000243] and 536 [CP000247].

### Analysis of the plasmid and chromosomal upstream α-*hly *operons

**Figure 2:**
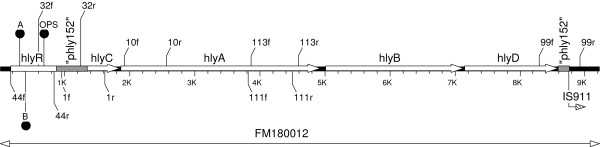
**Map of the α-*hly *region of plasmid pEO5 (**FM180012**)**. The positions of PCR-primers used for investigation of strains with plasmid and chromosomally inherited α-*hly *genes are indicated as leaders carrying the primer designations (Table 2). Regulatory sequences inside the *hlyR *gene (A, B and OPS) are shown as filled ballons. "phly152" is a stretch of non-coding DNA showing strong homology to corresponding regions in the α-*hly *plasmid pHly152.

Based on the pEO5 DNA sequence (Fig. 2) we developed specific primers for amplification of fragments within the *hlyR*, and *hlyR *- *hlyC *regions (Table 2). In addition, we developed specific PCRs for the upstream *hlyC *sequences of the chromosomal α-hemolysin operons in PAI I and PAI II of strain 536 [15] (Table 2). We performed PCR analysis of all strains carrying plasmid and chromosomal α-*hly *operons; strains carrying α-*hly*-plasmids pEO5 and pHly152 and 536 served as positive controls. The results are summarized in Table 1.

**Table 2:** Specific PCRs for identification of plasmid and chromosomally inherited α-*hly *determinants

DNA-target (position in sequence)	GenBank Accession	Primer	nucleotide sequence (5' - 3')	Tm (°C)	PCR product bp
*hlyA *(1915-1936) (2560-2580)	FM180012	10f 10r	GCTGCAAATAAATTGCACTCAG CCCTGCACCGATATTATCAAG	53.1	666
"pHly152" (953-974) &*hlyC *(1612-1630)	FM180012	1f 1r	GTAGTTCAAAAGACAACTCGTG ATCCCCGAAAGGAGCAATC	50.6	678
*hlyR *(597-618) & "pHly152" (1246-1267)	FM180012	32f 32r	GTCTTGCCGTACAATAATTTCC TCCGTTTAATGTCATAACTCGC	56.5	671^a^
*hlyR *(167-188) (830-851)	FM180012	44f 44	ATTCCAAGCGAAGTCCATCCCC CATAAAGCATGATGCCACCACG	66.5	685^a^
*hlyA *(3817-3839) (4497-4518)	FM180012	111f 111r	GATGGCACAAAAGCAACCGAAG TTCTCGCTTGAAGGCCACATCC	55.4	702
*hlyA *(3865-3883) (4592-4613)	FM180012	113f 113r	CTTGGTGGCGATGTTAAGG GACTCTTTTTCAAACCAGTTCC	53.5	749
*hlyD *(8297-8319) & IS*911 *(8925-8946)	FM180012	99f 99r	GCAGAATGCCATCATTAAAGTG CCATGTAGCTCAAGTATCTGAC	53.8	650
PAI I (536) (44506-44524) &*hlyC *(45278-45299)	AJ488511	81f 81r	CCTGTGACACTTCTCTTGC CCCAAGAACCTCTAATGGATTG	52.3	773^a^
PAI II (536) (31974-31995) &*hlyC *(32650-32668)	AJ494981	72f 72r	CCCAACTACAATATGCAACAGG CGCCAATAGAGTTGCCTTC	51.9	695

a) PCR products of different lengths were obtained with these primers depending on the DNA template (see Table 1)

Primers 1f/r are specific for the upstream *hlyC *region in pEO5 and yielded a PCR product of 678 bp (Fig. 2). PCR products of the same size were obtained with all strains carrying α-*hly *plasmids, except 84/S (pEO14); restriction enzyme analysis revealed all the fragments had a similar HinfI profile (data not shown). Primers 1f/r gave no products using *E. coli *strains carrying chromosomally encoded α-*hly *as template with the exception of the *E. cloacae *strain KK6-16 which yielded a PCR product; DNA sequencing revealed a 778 bp fragment [GenBank FM210352, position 72-849] (Table 1).

**Figure 3:**
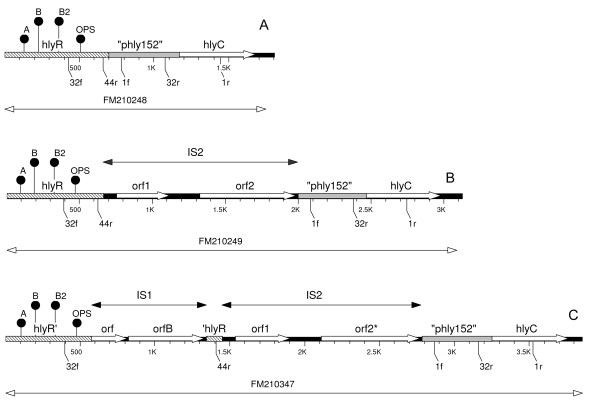
**Map of the *hlyR*-*hlyC *region of representative plasmids of groups 1, 2 and 3**. Genetic map of the corresponding regions from *hlyR *to *hlyC *of α-*hly *determinants from plasmids representing groups 1-3. A) pEO9, (strain 84-2195) B) pEO11, (84-3208); and C) pEO853 (CB853). The positions of PCR-primers used for identification and nucleotide sequencing are indicated as leaders carrying the primer designations (Table 2). Regulatory sequences inside the *hlyR *gene (A, B and OPS) are shown as filled ballons. "phly152" is a stretch of non-coding DNA showing strong homology to corresponding regions in the α-*hly *plasmid pHly152.

Primers 32f/r spanning the region between *hlyR *and the "phly152" segment amplify a 671 bp product in pEO5 [GenBank FM180012, position 597-1267] (Fig. 2). A PCR product of the same size was obtained with pEO5 and derivative plasmids as well as with plasmids pEO9 [GenBank FM210248 position 427-1097], pEO13 and pEO860 (Table 1, Fig. 3). Primers 32f/r yielded PCR products of 2007 bp with pEO11, [GenBank FM210249, position 392-2398), pHly152 and pEO12, and 2784 bp PCR products with pEO853 [GenBank FM10347 position 399-3182], pEO855 and pEO857 (Table 1). All amplicons of a given size (671 bp, 2007 bp and 2784 bp), yielded a similar HinfI restriction pattern (data not shown). Strains with chromosomally encoded α-hemolysin gave no products in the 32f/r PCR, as well as strain 84/2 S carrying plasmid pEO14 (Table 1).

Primer pair 44f/r (Fig. 2) is specific for *hlyR *and amplified related sequences in all *E. coli *carrying α-*hly *plasmids except pEO14. The *hlyR *PCR product was 685 bp (pEO5) [GeneBank FM180012, position 167-851] for all plasmids except for pEO853, pEO855, pEO857 and pEO860 which generated amplicons of about 1400 bp (Table 1). The 685 bp and 1400 bp size PCR products yielded similar HinfI restriction profiles, respectively. Strains with chromosomally inherited α-*hly *genes were negative for *hlyR *sequences (Table 1).

None of the strains with α-*hly *plasmids, or the *E. cloacae *strain KK6-16 yielded PCR products with primer pairs 81f/r and 72f/r, that are specific for PAI I and PAI II α-hemolysins (Table 2) [17]. All strains with chromosomal α-*hly *except KK6-16 produced PCR products with one or both of these primer pairs (Table 1). Taken together, the PCR typing indicated that all plasmid α-*hly *except pEO14 were similar for the regulatory regions located upstream of the *hly*-genes which differed from the chromosomal α-*hly *operons.

### Genetic analysis of the region between *hlyR *and *hlyC *in α-*hly *plasmids

A 464 bp DNA segment that carries a promoter (pHhly_L_) for expression of α-*hly*-genes is located directly upstream of the *hlyC *gene in plasmid pHly152 [24] [GenBank M14107]. A 466 bp region with 98.9% sequence homology was found upstream of *hlyC *in pEO5 [21]. The "phly152" region is not present in *E. coli *strains containing chromosomal α-*hly *genes [20] (this work). Sequences highly homologous to a large part of the "phly152" region were found in all α-*hly *plasmids investigated here, except pEO14. Comparison of the complete 466 bp "phly152" DNA stretch of plasmids pEO5 [GenBank FM180012], pEO9 [FM210248], pEO853 [FM210347], pEO11 [FM210249] and pEO860 [FM210351] revealed similarities from 97.9% to 100%. Interestingly, a 427 bp fragment with 93% similarity to the "phly152" segment was found upstream of *hlyC *in the *E. cloacae *strain KK6-16 [GenBank FM210352, position 1-427].

Sequences specific for *hlyR *[GenBank X07565], a regulatory region located about 2000 bp upstream of the α-*hly *determinant in pHly152 [28] were present in all α-*hly *plasmids except pEO14. The *hlyR *regions of five representative plasmids (pHly152, pEO5, pEO9, pEO11 and pEO853) were analyzed and compared to each other (Fig. 3). Short DNA sequences that were reported to be involved in regulation of α-*hly *expression located inside *hlyR*, i.e regulatory sequences A and B [28] and the "operon polarity suppressor (ops) [18], were identified in the corresponding *hlyR *region of the five plasmids. A clustal analysis performed with a 565 bp segment of the *hlyR *region beginning with the regulatory sequence A to the end of the *hlyR *region revealed 98.8 to 100% similarity between these five plasmids.

### IS*1 *and IS*2 *elements are present in the α *hly*-upstream region of plasmid *hly*

According to the length of PCR products obtained for the region located between *hlyR *and *hlyC *genes three groups of α-*hly *plasmids were established (Table 1). "Group 1" is represented by pEO5, its homologues from other *E. coli *O26 strains and by pEO9 and pEO13. "Group 2" is represented by pHly152, pEO11 and pEO12. "Group 3" is formed by plasmids pEO853, pEO855 and pEO857 from porcine strains. Two strains with α-*hly *plasmids pEO14 and pEO860 showed individual patterns by PCR-typing (Table 1). In order to explore the differences between the major groups of α-*hly*-plasmids we determined the nucleotide sequence of the region located between *hlyR *and *hlyC *of three representative plasmids, namely pEO9 [GenBank FM210248], pEO11 [FM210249] and pEO853 [FM210347] (Fig. 3).

Major differences between the α-*hly *plasmids in the region between *hlyR *and *hlyC *caused by insertion of IS*1 *and IS*2*. While "group 1" plasmids (pEO5, pEO9 and pEO13) carry no IS elements all "group 2" plasmids (phly152, pEO11 and pEO12) carry an IS*2 *element inserted directly downstream of the 3' end of *hlyR *(5' CCTGG 3') in pEO11. A 326 bp part of the IS*2 *element was previously described in pHly152 [GenBank M14107] [24], it is 99.4% identical to the corresponding IS*2 *element of pEO11. The IS*2 *elements in pEO11 and pHly152 are inserted at the DNA same site and are both flanked by the duplicated 5' CCTGG 3' DNA sequence.

Plasmids belonging to "group 3", which were all from pig strains (pEO853, pEO855 and pEO857), carry two IS elements in the region between *hlyR *to *hlyC*. In pEO853, the 786 bp IS*1 *is inserted immediately downstream of the *hlyR *internal sequence 5' AACAAAATT 3'. This 9 bp DNA stretch is repeated at the right hand end of the inserted IS*1 *and followed by the 94 bp residual 3' end of the *hlyR *region (Fig. 3). The IS*2 *element of pEO853 is 99.8% similar to that of pEO11 and inserted at the same position as in "group 2" plasmids pEO11 and pHly152.

### Investigation of *hlyR-hlyC *region of STEC strains of porcine origin

We used the primers specific for the region between *hlyR *to *hlyC *(Table 1) to investigate 26 α-hemolysin/*stx*2e STEC strains from diseased pigs or pork meat [29]. PCR products were obtained from all. According to the length of the amplicons generated with primers 1f/r, 32f/r and 44f/r all but one strain showed patterns indicating the presence of a "group 2" or "group 3" plasmid with IS-elements in the region between *hlyR *and *hlyC *(Table 3). The PCR-profiles were closely associated with serotypes of strains causing edema disease in pigs (O138:H14, O139:H1 and O141:H4) suggesting that α-*hly *plasmids are conserved in these strains.

**Table 3:** Detection of α-*hly *plasmid specific sequences in porcine STEC strains.

			Size of PCR products with primers^a^			
**Serotype**	**No. strains**	**Plasmid group**	**1f+1r**	**32f + 32r**	**44 f + 44r**	**99 f + 99r**

O138:H14^b^	4	3	678	2900	1500	650
O139:H1^b^	9	3	678	2900	1500	650
O139:H1^b^	1	single	678	2000	1500	650
O141:H4^b^	7	3	678	2900	1500	650
O141:H4^b^	3	2	678	2000	685	650
O2:H32^c^	1	2	678	2000	685	650
O36:H19^c^	1	2	678	2000	685	650

a) size of the PCR products (bp) with primers 1f/r, 32f/r, 44f/r and 99f/r as described in Tables 1+ 2. Digestion of PCR products by HinfI resulted in identical patterns between strains corresponding to the size of the PCR products obtained (Table 1)

b) Strains isolated from feces of diseased pigs [29]

c) Strains isolated from pork meat [29]

### Sequence analysis of the *hlyC *gene of plasmid and chromosomal α-hemolysin

**Figure 4:**
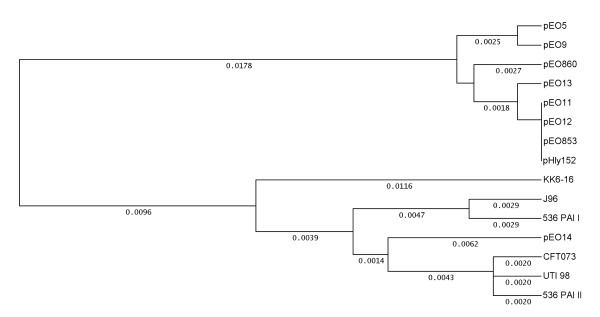
**Genetic relationship between plasmid and chromosomally inherited *hlyC *genes**. Clustal analysis of the coding sequence of the *hlyC *gene (513 bp) of strains 84-3208 (pEO11) [GenBank FM210249], 84-2 S (pEO14] [FM210350], 84-R (pEO13) [FM210348], 84-2195 (pEO9) [FM210248], C4115 (pEO5) [FM180012], CB860 (pEO860) [FM210351], CB853 (pEO853) [FM210347], 84-2573 (pEO12) [FM210349], KK6-16 [FM210352], 536 PAI I [AJ488511], 536 PAI II [AJ494981], CFT073 [AE014075], UTI98 [CP000243] and J96 [M10133]. UPGMA was used as tree building method and distances calculated according to Tajima and Nei 1984 [45].

The fact that α-*hly *plasmids were similar for the regulatory sequences upstream of the α-*hly *operon prompted us to analyze the coding sequence of seven plasmid *hlyC *genes, namely pEO9 [GenBank FM210248], pEO860 [FM210351], pEO13 [FM210348], pEO14 [FM210350], pEO11 [FM210249], pEO853 [FM210347], and pEO12 [FM210349] (Table 1). We used Clustal W analysis to compare the DNA sequences of the plasmid *hlyC *genes and the chromosomal *hlyC *genes of strain 536, PAI [GenBank AJ488511] and PAI II [AJ494981] UTI98 [CP000243], CFT073 [AE014075], J96 [M14107] and that of the *E. cloacae *strain KK6-16 [FM210352]. All plasmid *hlyC *sequences, except that of pEO14, showed 99.2 to 100% nucleotide sequence homology to each other and were grouped into one cluster (Fig. 4). A second cluster (98.5% to 99.6% similarity) was formed by the chromosomal and pEO14 *hlyC *genes (Fig. 4). The *hlyC *gene encoded by pEO14 was most similar to that of PAI II from strain 536 (99.2% homology). The *hly*C genes of all other α-*hly *plasmids showed 94.9-95.9% homology to chromosomal *hlyC *genes of *E. coli*. The amino acid (aa) sequences of *hlyC *translation products revealed five aa-exchanges (positions 3, 5, 40, 51, and 160) in the 170 aa-sequence that were closely associated with the origin (plasmid or chromosome) of the *E. coli hlyC *genes (data not shown).

The *hlyC *gene of the *E. cloacae *strain KK6-16 was more distant for its nucleotide and aa sequence from both the *E. coli *plasmid and chromosomal *hlyC *gene clusters and most similar to chromosomal PAI I, PAI II (98.2%) and pEO13 (97.2%) *hlyC *genes (Fig. 4).

### Comparison of nucleotide sequences of plasmid and chromosomal α-*hlyA *genes

Comparing the nucleotide sequences of *hlyA *revealed significant differences between chromosomal and plasmid genes. By BLAST search, a 755 bp stretch of the *hlyA *gene (position 3000-4095 in the pHly152 sequence, GenBank M14105) was found only 91% similar to the corresponding regions of the chromosomal *hlyA *genes from PAI I [AJ488511] and PAI II [AJ494981].

**Figure 5:**
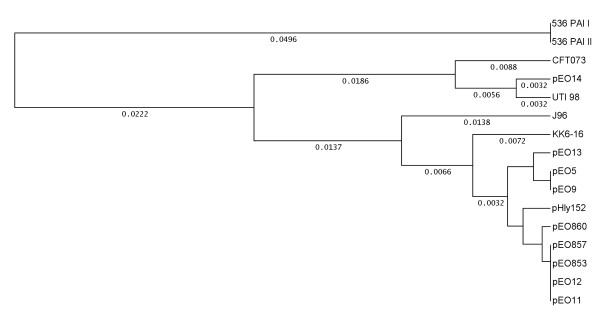
**Genetic relationship between plasmid and chromosomally inherited *hlyA *genes**. Clustal analysis of 633 bp internal *hlyA *sequence of strains 84-3208 (pEO11) [GenBank FN673696], 84-2 S (pEO14) [FN673697], 84-R (pEO13) [FN673698], 84-2195 (pEO9) [FN673699], C4115 (pEO5) [FM180012], CB860 (pEO860) [FN673700], CB853 (pEO853) [FN673701], CB857 (pEO857) [FN673702], 84-2573 (pEO12) [FN673703], KK6-16 [FN673704], 536 PAI I [AJ488511], 536 PAI II [AJ494981], CFT073 [AE014075], UTI98[CP000243] and J96 [M10133]. UPGMA was used as tree building method and distances calculated according to Tajima and Nei 1984 [45].

In order to explore the differences between plasmid and chromosomal *hlyA *genes we have developed PCR primers (111f/r and 113f/r from GenBank FM180012, Table 2) for amplification of this DNA region. The nucleotide sequence of the corresponding 633 bp PCR products from strains with α-*hly *plasmids and from *E. cloacae *strain KK6-16 was determined. The results are presented in Fig. 5. Except for pEO14, all plasmid encoded *hlyA *internal sequences were very similar to each other with a maximum difference of 1.4% (pHly152 and pEO13). In contrast, chromosomal *hlyA *genes showed differences of up to 9.5% when compared to each other (J96 compared to 536 both PAI I and PAI II). The 211 aa HlyA translation products showed aa-exchanges at positions 58 and 78 that were associated with the *E. coli *plasmid or chromosomal origin of the genes (data not shown).

The nucleotide sequence of the *hlyA *region on plasmid pEO14 was found closely related to the chromosomal *hlyA *gene of strain UTI98 (0.6% difference), and showed 5-6% sequence differences to all other α-*hly*-plasmids. Interestingly, the *E. cloacae hlyA *gene sequence was found 99% similar to that of plasmids pEO5 and pEO9 and more distantly related to the *E. coli *chromosomal *hlyA *genes (2.6 to 10.4% differences).

### IS*911 *is present downstream of *hlyD *in strains carrying α-*hly *plasmids

It was suggested that the *hlyCABD *operons were spread in *E. coli *by mobile genetic elements [20] and a truncated IS*911 *segment of 254 bp was found located closely and downstream of the *hlyD *gene in plasmid pEO5 [21]. In order to investigate the other α-*hly *plasmids for the presence of this element we developed PCR-primers (99f/r) encompassing a 650 bp stretch of DNA starting inside *hlyD *and ending inside the IS*911 *sequence. All α-*hly *plasmids except pEO14 yielded a PCR product. None of the strains carrying chromosomal α-*hly *genes reacted positive with this PCR (Table 1). The nucleotide sequence of the 579 bp amplicons from nine α-*hly *plasmids (strains CB860 [GenBank FN678780], CB857 [FN678781], CB853 [FN678782], 84-3208 [FN678783], 84-2573 [FN678784], 374 [FN678785], 84-R [FN678786], 84-2195 [FN678787] and CB855 [FN678788] were compared by Clustal W analysis. The sequences were 99.5-100% identical, indicating that the 3' part of the *hlyD *gene and downstream IS*911 *sequences are conserved among plasmid α-*hly *determinants. In addition, all 26 STEC strains from pigs or pork meat that carried α-*hly*-plasmids (Table 3) yielded 650 bp products with primers 99f/r, that showed similar HinfI digestion profiles (257, 222 and 171 bp) to those of the sequenced plasmids [FN678782-88] indicating that the *hlyD*-IS*911 *region is conserved in these strains.

### Transcriptional analysis of plasmid and chromosomal α-*hlyA *genes

We investigated if the presence of IS elements in the regulatory region upstream *hlyC *has an affect on transcription of the α-*hlyA *gene. Phenotypically, all strains with α-*hly *plasmids showed large and clear zones of hemolysis on blood agar plates similar to that found with strains carrying chromosomally inherited α-*hly *genes. An exception was made for strains 536-14 (the PAI I deletion mutant of strain 536) and the wildtype strain 695/83 (Table 1), which generated small, turbid zones of hemolysis on blood agar plates [19].

**Figure 6:**
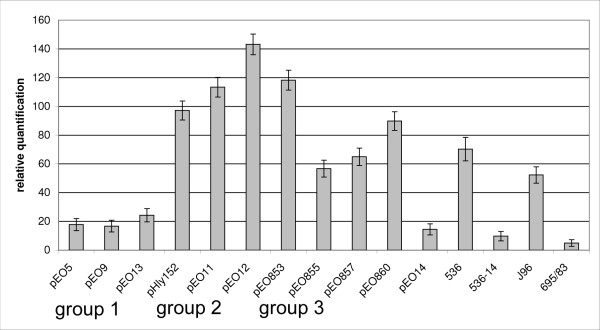
**Relative quantification of the *hlyA *gene transcription in *E. coli *strains encoding plasmid and chromosomally inherited α-*hly *determinants**. Strains and plasmids as well as plasmid groups are listed in Table 1. Means and standard deviations from two separate experiments performed in duplicate are shown.

We compared the transcriptional activity of 15 *E. coli *strains carrying plasmid and chromosomal α-*hly *operons by analyzing the mRNA transcription level of the α-*hlyA *gene in a relative quantification (rq) assay by Real-Time PCR. The *E. coli icdA *housekeeping gene was used as a standard (Fig. 6). Transcription of the *hlyA *gene was higher than *icdA *in all strains (rq 4.8 to 143.2). Relatively low *hlyA *transcription rates (rq 4.8 and 9.7) were found with poor hemolysin producing strains 536-14 and 695/83. Strains carrying "group 1" α-*hly *plasmids (pEO5, pEO9 and pEO13) as well as pEO14 showed significantly (95% confidence intervals) lower transcription rates (rq 14.4 -24.3) compared to "group 2" and "group 3" strains with IS elements inserted upstream *hlyC *(rq 56.7 to 143.2). Significant differences in *hlyA *transcription rates were found between individual strains carrying "group 2" and "group 3" plasmids but they could not be clearly assigned to one of two groups. Except for pEO12 and pEO853, all "group 2" and "group 3" strains showed *hlyA *transcription rates that were not significantly different from those of strains 536 and J96, the latter carry each two chromosomally inherited α-*hly *genes [16,17].

## Discussion

We have recently determined the nucleotide sequence of the pEO5 α-*hly *genes, which are commonly occurring in EPEC O26 strains from humans and animals [21]. Surprisingly, the α-*hly *genes were 99.2% similar to that of pHly152 which originates from a murine *E. coli *strain. Moreover, plasmid encoded α-*hly *genes from pHly152 and pEO5 showed close similarity in the regulatory region upstream of *hlyC *and thus differed from chromosomally inherited α-*hly *determinants [21]. These findings may indicate that plasmid encoded α-hemolysins have evolved from one source and separately from the chromosomal hemolysin operons. In order to explore this possibility we compared plasmid α-*hly *from unrelated *E. coli *strains of human, mouse, canine and porcine origin for similarities the regulatory and structural genes and their adjacent sequences.

Plasmid encoded α-*hly *determinants were found similar to each other in their genes (*hlyR*, *hlyC*, *hlyA *and *hlyD*) as well as in the adjacent sequences upstream and downstream of the α-*hly*-operon. Plasmid encoded *hlyC *and *hlyA *genes showed typical alterations in the nucleotide and in the amino acid sequence compared to their chromosomally encoded homologues. Moreover, chromosomally encoded α-*hly *genes were found different for the regions encompassing the α-*hly*-operon. The finding that chromosomal *hlyC *and *hlyA *genes clustered separately and showed greater sequence diversity compared to the plasmid homologues suggests that plasmid α-*hly*-genes have emerged more recently in *E. coli *and thus accumulated fewer changes compared to the chromosomal α-*hly *genes.

It was previously suggested that α-*hly *genes were acquired by strains of *E. coli *by horizontal gene transfer [25,27,30]. This hypothesis is supported by the location of chromosomally encoded hemolysin genes on pathogenicity islands [13,14,16,17] and the flanking of plasmid encoded α-*hly *genes by transposable elements [20,21]. A truncated IS*911 *element located downstream of the *hlyD *gene was found in all α-*hly *plasmids investigated in our study indicating that the plasmid encoded α-*hly *determinants may have descended from a common progenitor [31]. We do not know much about the genetic similarity between the α-*hly *plasmids investigated in this study, except that they show differences in size (48-157 kb) and conjugation ability. Further investigation of plasmid backbone sequences could reveal if they have descended from a common progenitor. At present, we cannot exclude that the α-*hly *determinant was transposed independently to different plasmids in *E. coli*.

Interestingly, plasmid pEO14 differed largely from all other α-*hly*-plasmids investigated in this study. The nucleotide sequence analysis of its α-*hly *genes and the adjacent sequences revealed close similarity to chromosomal α-*hly *genes. Because the α-*hl*y genes present on plasmid pEO14 shows all features of chromosomal α-*hly *operon it is likely that it was generated by recombination between a plasmid and chromosomal α-*hly *loci in *E. coli*. A similar event might have been involved in generation of a truncated α-*hly *segment in plasmid Vir68 that has been analyzed for its complete nucleotide sequence [GenBank CP001162].

The chromosomally located α-*hly *genes of the *E. cloacae *strain KK6-16 showed similarities to *E. coli *plasmid encoded α-*hly *determinants. The nucleotide sequence analysis of the 552 bp upstream region of the α-*hly *operon of KK6-16 was identical to that of the α-*hly *plasmid pANN681 [GeneBank L01627] [32] and the "pHly152" region found in all α-*hly *plasmids except pEO14 is present in KK6-16. Since α-*hly *is not common in strains of *Enterobacter *species [26], it seems likely that strain KK6-16 acquired the α-*hly *genes by conjugation from *E. coli*. Similar findings have been made for plasmids encoding antimicrobial resistance [33,34]. However, we have not investigated this possibility.

Interestingly, the *hlyC *and *hly*A sequences of the KK6-16 showed characteristic features which made it difficult to assign its α-*hly *determinant to the group of plasmid- or chromosomally inherited α-*hly *genes (Figs. 4+5). It is possible that characteristic alterations found in the KK6-16 α-*hly *sequence are due to *E. cloacae *as a different bacterial host species.

Multiple copies of IS*1 *and IS*2 *were frequently found in genetically unrelated strains of *E. coli*. IS*1 *and IS*2 *were found to be non-randomly scattered in the genomes of wild-type *E. coli *strains [35-37]. IS-elements are involved in chromosomal rearrangements, integration of F-plasmids and transposition of genes [38] and thus could have been involved in the generation of *E. coli *α-*hly *plasmids. Activation of downstream genes by presence of IS*1 *and IS*2 *elements in *E. coli *has been reported [39] and this could explain the relatively high *hlyA *transcription rates in plasmids carrying IS*2 *or IS*1 *and IS*2*. However, we have not tested this possibility experimentally and other factors such as plasmid copy numbers and differences between the *E. coli *host strains could have an influence on the transcription rates.

α-hemolysin plasmids are frequently found in STEC strains producing Stx2e, agents of edema disease in pigs [40], and in ETEC strains producing heat-stable enterotoxin causing diarrhea in dogs [10]. The α-*hly *plasmid pEO5 is closely associated with EPEC O26 strains as diarrheal agents of human infants and calves [21,41]. In contrast, *E. coli *strains carrying chromosomal α-*hly *are associated with UPEC which are characterized by other virulence attributes and serotypes than ETEC, EPEC and STEC strains [13,14,16,17]. The association of α-*hly *plasmids with intestinal and of chromosomal α-*hly *determinants with extraintestinal strains points to a separate evolution in these two major groups of pathogenic *E. coli*.

## Conclusion

Our results indicate that the α-*hly *genes present on plasmids in ETEC, STEC and EPEC strains have a common origin. The presence of IS-sequences flanking the plasmid α-*hly *genes suggest that these were introduced in *E. coli *by horizontal gene transfer. Plasmids were shown to play a role in the spread of α-*hly *determinant to *Enterobacter cloacae*. Chromosomally α-*hly *genes present in UPEC are genetically more diverse and seem to have evolved separately from the plasmid α-*hly *genes.

## Methods

### Bacteria

The bacterial strains used in this work are listed in Table 1. Strain C4115, the source of the plasmid pEO5, the *E. coli *strain 374 carrying the α-*hly*-plasmid pHly152 as well as other EPEC O26 strains were described previously [21]. The relevant characteristics of strains with chromosomally located α-hemolysin determinants are listed elsewhere [10,18,19]. The α-hemolytic *E. cloacae *strain KK6-16 as well as the canine and porcine ETEC and STEC strains carrying α-*hly *plasmids were described previously [10,26,29,42]. The EHEC-hemolysin plasmid pO157 carrying strain TPE1313 was used as negative control is described elsewhere [21]. Mating of bacteria with *E. coli *K-12 recipient strains and isolation of α-hemolytic transconjugants was performed as described by Burgos et al. 2009 [21]. Phenotypes corresponding to *E. coli *α-hemolysin were analyzed on washed sheep blood agar [43].

### Isolation of DNA, RNA and cDNA

Total DNA of bacteria was isolated as described [29]. Purified plasmid DNA of bacteria that was used for restriction digestion, DNA-hybridization, PCR and nucleotide sequencing was isolated with the large construct kit following the instructions of the producer (Qiagen, Hilden, Germany). Analysis of total plasmid profiles of *E. coli *strains was performed as described previously [44]. Total RNA was isolated from 20 ml of exponentially growing aerated cultures (3-5 × 10^8 ^bacteria/ml) of bacteria in L-Broth with the RNeasy minikit (Qiagen). Isolation of RNA and preparation of cDNA was performed as described previously [29].

### DNA hybridization

Southern blot hybridization of plasmid DNA and labeling of gene probes with Digoxigenin-11-dUTP was performed as described [21]. Dig-labeled molecular markers (Dig Roche) were used for size determination of hybridizing DNA fragments. For identification of α-*hly *plasmids in Southern blotted gels a 666 bp PCR product of the α-*hlyA *gene generated with primers 10f/r (Table 2) was used as internal DNA probe for detection of α-*hly *specific sequences [21]. Plasmids pHly152, pO157 and pEO5 served as reference plasmids for size determination of α-*hly *plasmids [21] (Fig. 1).

### Nucleotide sequencing of α-hemolysin and associated sequences

Nucleotide sequence analysis of the α-*hly *determinants and adjacent sequences was performed as described [21]. PCR products were purified and used for sequencing applying the dye terminator chemistry (PE Applied Biosystems, Darmstadt, Germany) and separated on an automated DNA sequencer (ABI PRISM^® ^3100 Genetic Analyzer, Applied Biosystems, Foster City, CA). The sequences were analyzed using the Lasergene software (DNASTAR, Madison,WI) and Accelrys Gene v2.5 software.

### Development of specific PCRs for plasmid- and chromosomally inherited α-*hly *determinants and their associated sequences

Primer pairs specific for α-*hly*-plasmid specific sequences *hlyR *(primers 44f/r), the region between *hlyR *and *hlyC *(primers 1f/r, 32f/r), *hlyA *(111f/r and 113f/r) and *hlyD *and downstream (99f/r) (Table 2) were developed with Accelrys software using the pEO5 sequence [GenBank FM180012]. Primer pairs for amplification of regions upstream of *hlyC *in chromosomally inherited α-hemolysins were developed from sequences of PAI I (81f/r) and PAI II (72f/r) from the uropathogenic *E. coli *strain 536 (Tables 1+2). Primers 10f/r served as positive control for general detection of plasmid and chromosomally inherited α-*hly *determinants. Primers and PCR conditions are listed in Table 2. PCR reactions were performed as described previously [29].

### Transcriptional analysis of α-*hlyA *genes by qRT-PCR

Quantitative real time reverse transcription PCR (qRT-PCR) was performed with the Applied Biosystems 7500 real time PCR system (Applied Biosystems) with cDNA samples from bacteria (see above). Transcription rates of the α-*hlyA *gene were compared to those of the *icdA *housekeeping gene. Primers hlyA-f 5' ACCTTGTCAGGACGGCAGAT 3' and hlyA-r 5' CCGTGCCATTCTTTTCATCA 3' and the VIC labeled TaqMan MGB probe 5' ACTGGGAATTGAAGTCC 3' were used for amplification of the α-*hlyA *gene. The primers and the gene probe for detection of the *icdA *gene were described recently [29]. Real time PCR amplification were performed in an "icdA & α-hlyA" multiplex assay and were analyzed with the 7500 system SDS software version 1.4 as described [29].

### GenBank accession numbers

The following nucleotide sequences derived from the α-hemolysin producing strains and α-*hly *plasmids from Table 1 were submitted to GenBank: strain 374 (pHly152) [GenBank FN678785]; 84-2195 (pEO9) [GenBank FM210248, FN673699, FN678787]; 84-3208 (pEO11) [GenBank FM210249, FN678787, FN673696]; CB853 (pEO853) [GenBank FM210347, FN678782, FN673701]; 84-R (pEO13) [GenBank FM210348, FN678786, FN673698]; 84-2573 (pEO12) [FM210349, FN678784, FN673703]; 84-2 S (pEO14) [GenBank FM210350, FN673697]; CB860 (pEO860) [GenBank FM210351, FN678780, FN673700]; CB855 (pEO855) [GenBank FN678788]; CB857 (pEO857) [GenBank (FN678781, FN673702] and strain KK6-16 [FM210352, FN673704].

## Authors' contributions

LB took an integral part of project conception and both YB and LB in method development. YB took most part in the design and performance of the experimental procedures. Data analysis was performed by both researchers LB and YB as well as interpretation of results and preparation of the manuscript. All authors have read and approved the final manuscript
